# Resveratrol in Intestinal Health and Disease: Focusing on Intestinal Barrier

**DOI:** 10.3389/fnut.2022.848400

**Published:** 2022-03-16

**Authors:** Youxia Wang, Changming Hong, Zebiao Wu, Shuwei Li, Yaoyao Xia, Yuying Liang, Xiaohua He, Xinyu Xiao, Wenjie Tang

**Affiliations:** ^1^College of Animal Science, South China Agricultural University, Guangzhou, China; ^2^Animal Breeding and Genetics Key Laboratory of Sichuan Province, Sichuan Animal Science Academy, Chengdu, China; ^3^Livestock and Poultry Biological Products Key Laboratory of Sichuan Province, Sichuan Animtech Feed Co., Ltd., Chengdu, China

**Keywords:** resveratrol, intestinal barrier, antioxidant, anti-inflammation, anti-tumor

## Abstract

The integrity of intestinal barrier determines intestinal homeostasis, which could be affected by various factors, like physical, chemical, and biological stimuli. Therefore, it is of considerable interest and importance to maintain intestinal barrier function. Fortunately, many plant polyphenols, including resveratrol, could affect the health of intestinal barrier. Resveratrol has many biological functions, such as antioxidant, anti-inflammation, anti-tumor, and anti-cardiovascular diseases. Accumulating studies have shown that resveratrol affects intestinal tight junction, microbial composition, and inflammation. In this review, we summarize the effects of resveratrol on intestinal barriers as well as the potential mechanisms (e.g., inhibiting the growth of pathogenic bacteria and fungi, regulating the expression of tight junction proteins, and increasing anti-inflammatory T cells while reducing pro-inflammatory T cells), and highlight the applications of resveratrol in ameliorating various intestinal diseases.

## Introduction

**Figure 1:**
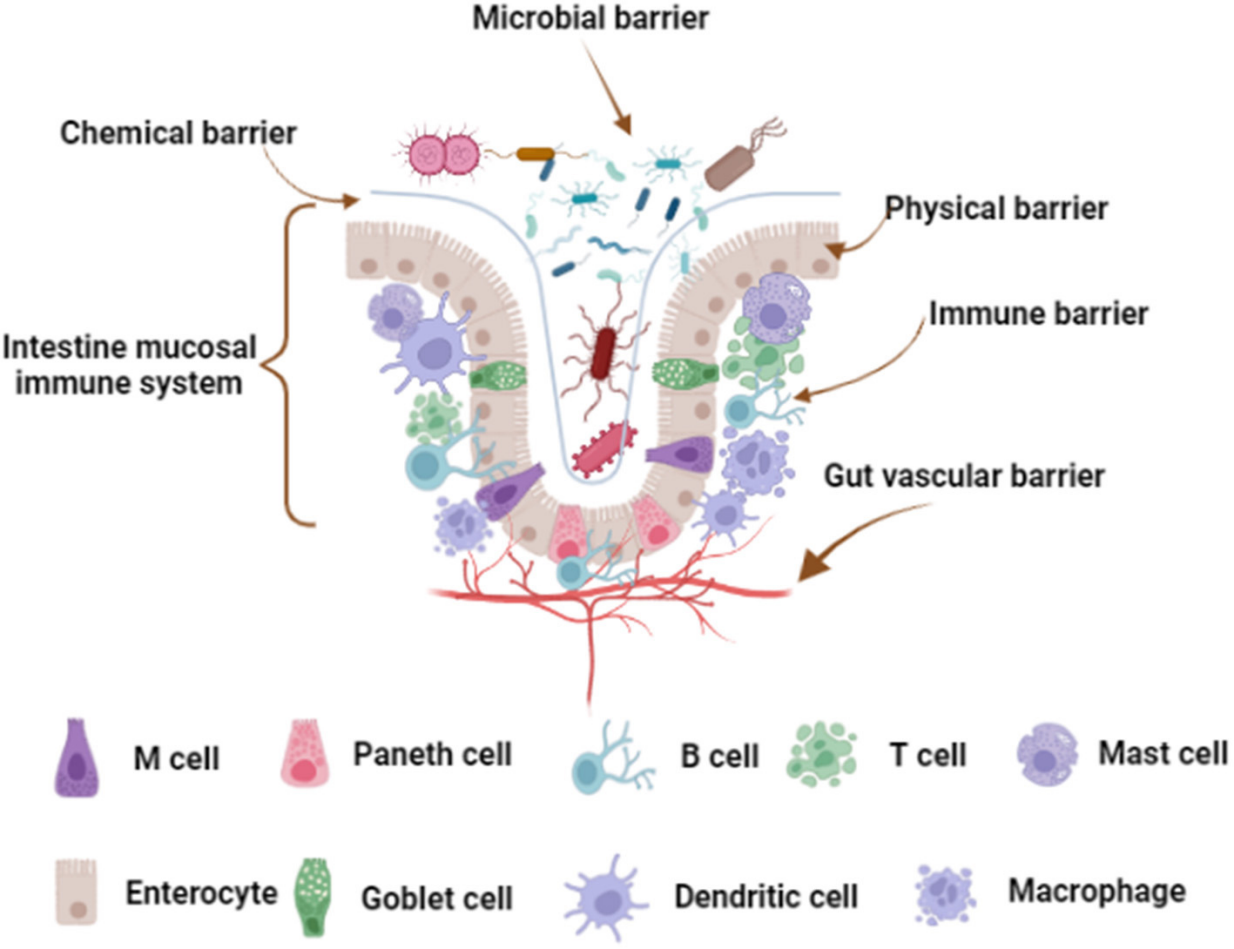
Intestinal barrier composition. The microbial barrier is the uppermost barrier of the intestinal tract. The chemical barrier is close to the microbial barrier and is a layer of mucous membrane. Physical barrier, also known as mechanical barrier, has mucosal epithelium, lamina propria and muscularis mucosae. Mucosal epithelium has an inherent epithelium, including immune cells and enterocytes, and there are tight junctions between the cells. The immune barrier is located below the lamina propria, including various immune cells scattered in the LP and MLNs. The gut vascular barrier is the deepest protective layer of the intestinal tract. The figure was created with Biorender.com.

Gut is the main organ for digestion and absorption of nutrients, almost 95% of the nutrients are absorbed by small intestine (1, 2). Gut is the largest microecosystem of the body that exists 1,000 different bacterial species, as evidenced by the quantity reaches 10^14^ and 100-fold more genes than that found in the human genome (3). Notably, the gut is also considered to be the largest immune organ, which has a strong mucosal immune system and contains the largest library of immune cells in the body (4). Most importantly, the intestinal barrier, including microbial, chemical, physical, immune, and gut vascular barrier, plays a vital role in protecting intestinal health (Figure 1) (5). The rich species of microorganisms constitute the intestinal microbial barrier. Indeed, the largest number of phyla in the intestinal tract is *Firmicutes*, about 65%; the second is *Bacteroidetes*, about 25%; and a small number of *Actinobacteria, Proteobacteria, Fusobacteria*, and *Verrucomicrobia* (6, 7). The chemical barrier consists of mucus, digestive juice, and bacteriostatic substances (8). The physical barrier, also known as mechanical barrier, has mucosal epithelium, lamina propria, and muscularis mucosae, which can prevent the invasion of bacteria and macromolecules (9). The mucosal epithelium has an orderly arrangement of epithelial cells. Actually, there are tight junctions, adhesive junctions, and desmosomes between adjacent epithelial cells. The intestinal immune barrier is mainly composed of lymphocytes and immune cells in mesenteric lymph nodes (MLNs) and intestinal lamina propria (LP) (10). In addition, gut is called “the second brain” of the host. The number of neurons distributed in the intestinal tract is second only to the brain, with about 100 million nerve cells, far more than the spinal cord and peripheral nervous system (11). Recently, study shown that gut vascular barrier (GVB) is the deepest protective layer of the intestinal tract, this is the last barrier that prevents microorganism and harmful toxins cross into the circulatory system and peripheral organs (12). Therefore, fine-tuning of intestinal health is the first element for body health.

In recent years, studies have found that many host diseases are related to intestinal health (13–15); and dietary polyphenols play important roles in regulating intestinal health (16–18). Resveratrol is a natural plant polyphenol, which was first isolated and purified from *veratrum grandiflorum* in 1940, and is widely found in grape, polygonum cuspidatum, berry, and peanut (19). Resveratrol has both *cis* and *trans* structures, and mainly exists in *trans* structure in the nature (20). Of note, resveratrol has many biological functions, such as antioxidant, anti-inflammation, anti-tumor, and anti-cardiovascular diseases, and with the particular interest to this article—protecting intestinal health (21–25). Notably, unless otherwise stated, all mentioned in the review are *trans*-resveratrol, and the *trans*-resveratrol is mainly grape extract. Herein, in this review, we summarize the effects of resveratrol on intestinal barrier, and highlight the applications of resveratrol in ameliorating various intestinal diseases.

## Absorption and Metabolism of Resveratrol

**Figure 2:**
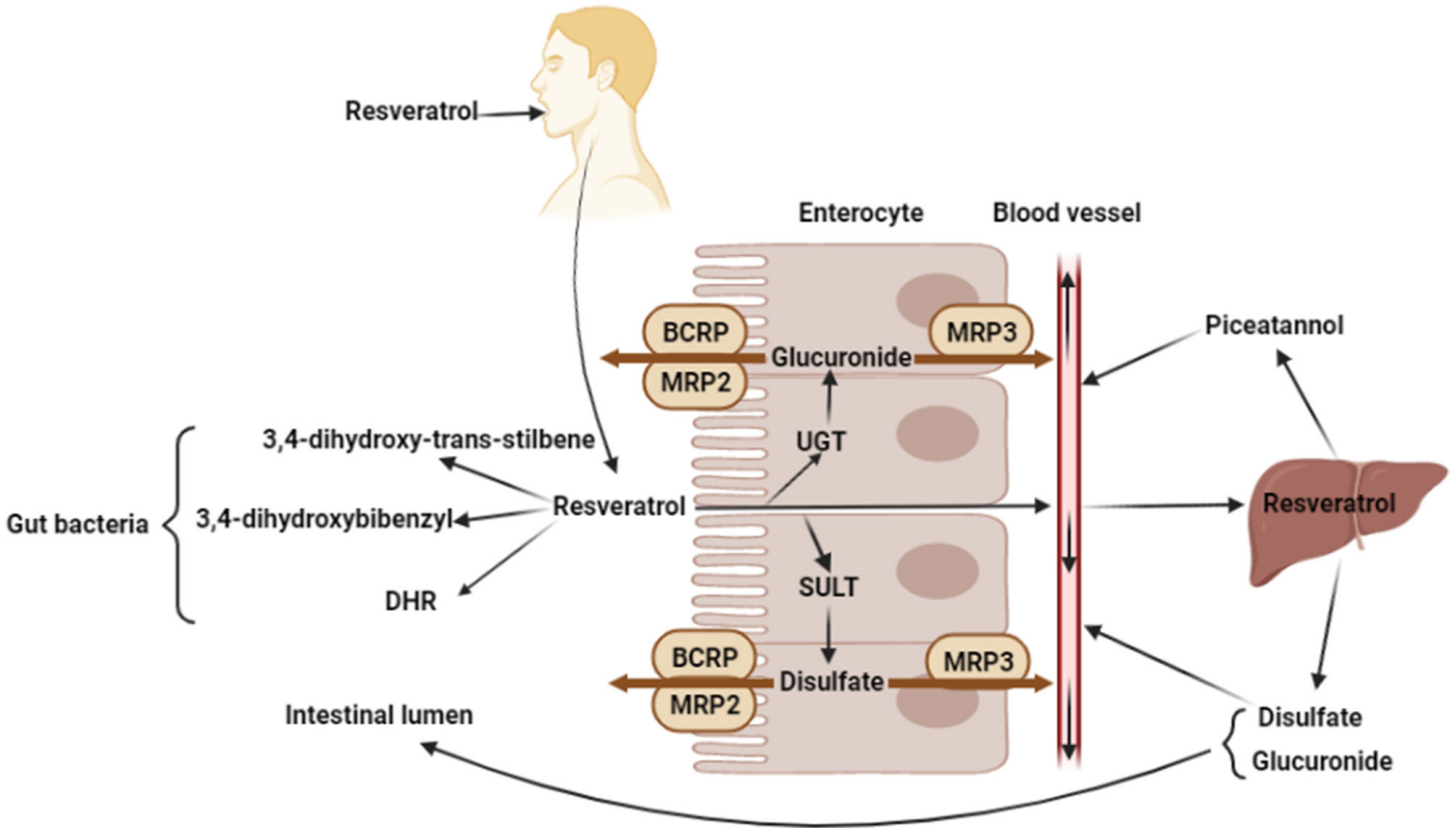
Absorption and metabolism of resveratrol. Oral resveratrol reaches the intestine and enters the enterocytes through passive diffusion. A small part of resveratrol can directly enter the blood circulation, while most resveratrol produces glucuronide and disulfate by the UDP-glucuronosyltransferases (UGT) and sulfotransferases (SULT) in enterocytes, and glucuronide and disulfate are transported back to intestinal lumen by the ABC transporter of enterocyte apical membrane (BCRP, MRP2), or transported into bloodstream by the ABC transporter of enterocyte basolateral membrane (MRP3). In addition, resveratrol produces 3,4-dihydroxybibenzyl, 3,4-dihydroxy-trans-stilbene, and dihydroresveratrol (DHR) by microorganisms. And in liver, resveratrol is metabolized to produce piceatannol, disulfate and glucuronide. The figure was created with Biorender.com.

The molecular weight of resveratrol is 228.25 g/mol, and resveratrol has benzene ring, hydroxyl groups, and C-C double bond, that could affect water solubility and absorption in intestinal tract (26, 27). In the intestinal tract, there are two ways to absorb and utilize resveratrol, including the absorption by enterocytes and the utilization by bacteria (Figure 2).

Resveratrol is perceived as a xenobiotics by gastrointestinal tract and crosses the intestinal epithelium to the blood through transcellular pathway (28). In the intestine, resveratrol binds to the proteins and lipids which will influence its absorption or elimination in feces (27). Enterocytes play an important role in the transcellular pathway absorption of resveratrol, which enters enterocytes through passive diffusion and forms complexes with intestinal membrane transporters (29). The ATP-binding cassette (ABC) family is one of the largest transporter families of lipid membranes. So far, 49 ABC transporter subtypes have been identified, which can be divided into 7 subfamilies (30, 31). Oral resveratrol reaches the intestine and enters the enterocytes through passive diffusion. Then, resveratrol is metabolized or directly into the bloodstream. However, only a small part of resveratrol can directly enter the blood circulation and be transported to peripheral organs, for example, the liver. Most resveratrol produces glucuronide and disulfate by the UDP-glucuronosyltransferases (UGT) and sulfotransferases (SULT) in enterocytes (32), and glucuronide and disulfate are transported back to intestinal lumen by the ABC transporter of enterocyte apical membrane (BCRP, MRP2), or transported into bloodstream by the ABC transporter of enterocyte basolateral membrane (MRP3) (33, 34). Glucuronide and disulfate are the major circulating forms of resveratrol, which could be detected in liver, adipose tissue, or the heart, after oral administration (35, 36). Resveratrol or its metabolites enter the bloodstream and bind proteins and fats to circulate throughout the body. In addition, resveratrol entering the bloodstream can be metabolized in the liver, for instance, resveratrol is hydroxylated to form piceatannol by the liver cytochrome P450 (34, 37, 38). Besides, resveratrol, which is not passively absorbed by intestinal epithelial cells, would be degraded by some intestinal bacteria. *Slackia equolifaciens* and *Adlercreutzia equolifaciens* have been proved to convert resveratrol to dihydroresveratrol (DHR) through hydrogenation (39). Another study shown that resveratrol can be metabolized by gut bacteria into other metabolites, 3,4-dihydroxy-trans-stilbene and 3,4-dihydroxybibenzyl (lunularin). However, the exact bacteria that produce these two metabolites are still unknown (40). Besides, the piceid, which is resveratrol precursor, is metabolized to produce dihydropiceid and DHR by the gut bacteria (41).

## Resveratrol and Intestinal Barrier

The intestinal barrier, which is useful for nutrient absorption and protection from external invasion, is composed of five layers: microbial barrier, chemical barrier, physical barrier, immune barrier, and gut vascular barrier (42). In this section, we discuss the associated investigations about how resveratrol affects the aformentioned intestinal barriers.

### Microbial Barrier

The rapid metabolism in the intestinal tract and the characteristic of poor water solubility may lead to low bioavailability of resveratrol. After oral administration of resveratrol, 18% of resveratrol enters into the bloodstream and 25% of resveratrol is excreted, while the rest could be metabolized by liver, intestine, and gut microbiota (29). In addition, some gut microbiota could use resveratrol precursors to produce resveratrol and its derivatives (41, 43). In recent years, many studies have shown that resveratrol and its derivatives could modulate gut microbiota composition and affect gut barrier function.

Resveratrol performs its antibacterial and antifungal activities through inhibiting the growth of bacteria and fungi (44). Resveratrol inhibits the growth of *Vibrio cholerae* through inhibiting the formation of its biofilm, and the minimum inhibitory concentration (MIC) is 60 μg/ml, which is dose-dependent (45). Resveratrol also inhibits the growth of *Campylobacter jejuni* and *Campylobacter coli* (which are the major cause of bacterial gastroenteritis) through the formation of its biofilm, and the MIC are 100 and 50 μg/ml, respectively (46). Moreover, resveratrol inhibits the growth of *Escherichia coli* in three ways. First, resveratrol binds reversibly to ATP synthase of *Escherichia coli* that affects the ATP hydrolysis and synthesis. Second, resveratrol induces DNA fragmentation and concomitant upregulation of the stress-response regulon in *Escherichia coli*. Finally, resveratrol treatment has been correlated with membrane damage (47, 48). Resveratrol also has inhibitory effect on other Gram-positive pathogens, such as *S. aureus, Enterococcus faecalis*, and *Streptococcus pyogenes*, and the MIC is 100–200 μg/ml (44).

Additionally, resveratrol also has inhibitory effect on some fungi, such as *Candida albicans, Saccharomyces cerevisiae*, and *Trichosporon beigelii*, and the inhibitory activity is 10–20 μg/ml (49). Interestingly, in weaned piglets, resveratrol exhibits significant inhibiting ability against *Enterococcus* and *Clostridium* (50). However, the inhibitory effect of resveratrol on *Candida albicans* remains controversial. Collado-González and Weber et al. failed to detect the antifungal effect of resveratrol against *Candida albicans* (51, 52). Intriguingly, Houillé et al. (53) found that resveratrol derivates has inhibiting effect on *Candida albicans* and *non-albicans Candida* (NAC) species in structure- and/or dose-dependent manner. Afterward, studies have revealed that resveratrol could induce *Candida albicans* apoptosis and have synergistic effect on azoles (an antifungal medicine) against *Candida albicans* (54, 55). These findings suggest that it might be structure- and dose-dependent on the antimicrobial effect of resveratrol and its derivates. Nevertheless, the above findings still need experimental confirmation.

Resveratrol not only inhibits the growth of intestinal pathogens, but also affects the abundance of intestinal dominant flora. Studies have found that resveratrol increases the abundance of *Bacteroides, Lactobacillus*, and *Bifidobacterium* in the intestines of mice and rats (56, 57). In DSS-induced colitic mice model, dietary resveratrol significantly enriches the gut microbiota, and restores bacterial community diversity and rebalances the probiotics and pathobionts (58). In db/db mice, resveratrol treatment decreases the relative abundance of *Firmicutes* and increases abundance of *Bacteroidetes*. Importantly, *Bacteroides, Alistipes*, and *Parabacteroides*, which exhibit anti-inflammatory properties, are markedly increased in the resveratrol-treated db/db mice group (59). In high-fat diet (HFD)-fed mice, resveratrol treatment significantly increases *Lactobacillus* and *Bifidobacterium*, whereas *Enterococcus faecalis* is significantly decreased, and resveratrol supplemented diets cause a higher abundance of *Bacteroidetes* while a lower abundance of *Firmicutes* (60). Additionally, the number of *Lactobacillus* and *Bifidobacterium* is significantly increased in resveratrol-fed animals (61).

Together, resveratrol improves intestinal microbial barrier by inhibiting the growth of pathogenic bacteria and fungi and modulating the composition of intestinal dominant flora. Although the mechanism of resveratrol in inhibiting the growth of *Escherichia coli* has been identified, the inhibitory effects of resveratrol on other pathogens still need to be further explored.

### Chemical Barrier

Intestinal chemical barrier plays an important role in resisting and killing pathogenic bacteria. Various mucins of intestinal chemical barrier can protect intestinal epithelia cells from pathogen invasion (62). Resveratrol has been found that it well orchestrates the intestinal chemical barrier. For instance, oxyresveratrol significantly increases the expression of mucin 2 (MUC2) through the increasing level of NAD+ in human LS174 goblet cells (63, 64). Moreover, the mRNA level of trefoil factor 3 (TFF3), which could increase mucosal integrity and mucus viscosity, is also increased (63). Subsequently, a recently study demonstrated that oxyresveratrol triggers the endoplasmic reticulum (ER) stress and promotes the expression levels of autophagy-related genes and ultimately induces MUC2 formation in human LS174 goblet cells (65). The increasing expression of MUC2 and TFF3 also occurs in the HFD-induced mice with resveratrol treatment (66).

Collectively, resveratrol is helpful for the maintenance and repairment of intestinal chemical barrier via increasing the expression and secretion of mucin. The possible mechanism is largely associated with the triggering ER stress in goblet cells by resveratrol treatment. Additionally, it is worthy of exploring whether there are other links such as bile salts and mucoitins between resveratrol and intestinal chemical barrier.

### Physical Barrier

The physical barrier is the largest and most important barrier in the intestine, and the tight junction (TJ) (e.g., ZO-1, ZO-2, and occludin) is the most important factor that contributes to the integrity of intestinal physical barrier (67). In the model of cyclophosphamide-induced immunosuppressed mice, the expression of ZO-1, ZO-2, and occludin proteins are significantly decreased, whereas resveratrol treatment can increase the expression of those TJ-associated proteins (68). Another study found that the mRNA levels of TJ-associated proteins were down-regulated in HFD-induced mice, which can be reversed after resveratrol treatment (66). In addition, resveratrol can prevent porcine intestinal epithelial cells from deoxynivalenol-induced damage through the Nrf2 signaling pathway (69), and decrease radiation-induced damage in intestinal epithelial cells via facilitating autophagy and preventing apoptosis by the activation of SIRT1 (70). Meanwhile, resveratrol can alleviate H_2_O_2_-induced damage through upregulating the expression of tight-junction proteins (occludin, claudin-1, and ZO-1), which depends on the PI3K/Akt-mediated Nrf2 signaling pathway (71).

In conclusion, resveratrol could protect the intestinal physical barrier from damage, which might be related to the regulation of tight junction protein expression and mitigation of oxidative stress.

### Immune Barrier

The intestinal immune barrier plays a vital role in protecting body health. Indeed, there are a large number of immune cells and lymphocytes in the MLNs and LP, which play important roles in the intestinal immunity (72). Resveratrol is an antitoxin produced by plant, and its anti-inflammatory function has been well documented. Several studies have shown that resveratrol could improve intestinal immune barrier. For example, resveratrol affects intestinal immune cells and lymphoid tissue. In colitis mice, resveratrol treatment increases the number of anti-inflammatory regulatory T cells (CD4+FOXP3+ and CD4+IL-10+) and down-regulates the number of inflammatory T cells, such as Th1 (CD4+IFN-γ+) and Th17 (CD4+IL-17+) cells in MLNs (73). In IL-10(–/–) chronic colitis mice, resveratrol treatment decreases the quantity of CXCR3+ T cells in MLNs and LP, and increases the percentage and absolute numbers of CD11b+ and Gr-1+ myeloid derived suppressor cells (MDSCs) in LP (74). Low dose resveratrol regulates Treg/Th17 balance through reducing the number of Th17 cells, while high dose resveratrol shapes Treg/Th17 balance through down-regulating the number of Th17 cells and up-regulating the number of Treg cells in ulcerative colitis mice (75). Moreover, resveratrol facilitates Th1/Th2 balance towards Th2 polarization and enhances Treg/Th17 balance towards Treg in the small intestine LP in mice (76). Intriguingly, in colitis mice, after resveratrol treatment, the percentage of CD4+ T cells in MLNs is restored to normal level, but decreases these cells in the colon LP. Likewise, the percentages of macrophages in MLN and the LP of colitis mice are decreased after resveratrol treatment. And resveratrol reverses the increased levels of tumor necrosis factor-α (TNF-α), interleukin (IL-6), and interleukin 1β (IL-1β) (77). Furthermore, resveratrol could raise the IgG concentration in serum of weaning piglets (78). Moreover, resveratrol inhibits degranulation and expression of CXCL8, CCL2, CCL3, and CCL4 in a dose-dependent manner in human intestinal mast cells. Resveratrol blocks the phosphorylation of extracellular signal-regulated kinase (ERK) 1/2 and signal transducer and activator of transcription (STAT) 3. Mitochondrial STAT3 is phosphorylated by ERK1/2 and contributes to mast cell degranulation (79). In OVA-induced mouse model, the percentage of mast cells is significantly increased in the MLNs, which could be reversed by resveratrol treatment (80).

Taken together, resveratrol increases anti-inflammatory T cells while reduces pro-inflammatory T cells. In addition, resveratrol also affects the number of macrophages and mast cells. Most importantly, resveratrol inhibits mast cell degranulation by blocking the phosphorylation of ERK1/2. Therefore, resveratrol can improve the intestinal immune barrier.

### Gut Vascular Barrier

The gut vascular barrier (GVB) is composed of intestinal vessel endothelium, which is an important valve to control the entry of pathogenic microorganisms and molecular substances into bloodstream, liver, brain, and other organs (12, 81). GVB has many characteristics in common with the blood-brain barrier (BBB), but GVB has more tolerant permeability, which allows for the diffusion of larger molecules (up to 4 kDa) (82). Therefore, if molecules (>4 kDa) and microorganisms cross mucous membrane and epithelial barrier, it will retain the LP, unless GVB is destroyed resulting in a change in permeability (83). Studies have shown that resveratrol reduces vascular oxidative stress, relieves vascular inflammation, and improves vascular function (84, 85). Besides, resveratrol decreases small intestinal pro-inflammatory cytokines and gut vascular permeability, which could attenuate the blood levels of pro-inflammatory cytokines from small intestine and alleviate cytokines-mediated BBB disruption and neuroinflammation (76). Thus, resveratrol may affect intestinal vascular permeability and improve GVB, but more researches are needed to prove the aforementioned function by resveratrol.

## Resveratrol and Intestinal Diseases

Given the above, the intestine is not only important for nutrition absorption, but also a key line of defense against the invasion of exogenous pathogenic microorganisms, and the damage of intestinal barrier is closely related to many diseases (86). Resveratrol has a positive effect on all intestinal barriers, this means that resveratrol can regulate many diseases related to intestinal barrier damage. Subsequently, in this part, we continue to explore the effects of resveratrol on several intestinal diseases (Table 1).

**Table 1:** Resveratrol and intestinal diseases.

**Disease**	**Species**	**Disease induction**	**Dose of RES, duration**	**Effects**	**References**
IBD	Rats	Irradiated	20 mg/kg/day, 3 weeks	Alleviated intestinal oxidative stress, reduced intestinal inflammation	(90)
		TNBS	2 or 10 mg/kg, 7 days	Alleviated ulceration and suppressed inflammation	(92)
		Heat-stressed	100 mg/kg/day, 3 days	Reduced intestinal injury and dysfunction	(89)
	Mice	DSS	100 mg/kg/day, 14 weeks	Decreased the inflammatory cytokine level	(91)
CRC	Human		0.5 or 1 g/day, 8 days	Reduced tumor cell proliferation by 5% and produced adequate resveratrol in the gastrointestinal tract to elicit anti-carcinogenic effect	(111)
		DMH	100 μM	Suppressed colon carcinogenesis at various stages via against NF-κB signaling pathway	(108)
			5 μM, 14 days	Down-regulated CRC cell survival, colony formation, invasion and activation of CSC cells	(110)
CRC	Mice		69.6 μM, 77.2 μM	Suppression of the invasion and metastasis of colon cancer	(109)
IBS	Mice	CACS	2.5, 5, 10 mg/kg	Reversed CACS-induced depression- and anxiety-like behaviors and intestinal dysfunction	(117)
	Rats	CACS	10 mg/kg	Improved anti-IBS-like effects on depression, anxiety, visceral hypersensitivity and intestinal motility abnormality	(118)
Bacterial infection	Avian	Avian pathogenic, *Escherichia coli*	128 μg/ml	Inhibition of APEC biofilm formation *via* regulating the levels of chemotaxis proteins	(123)
	Human	*Campylobacter jejuni*	100 μM	Alleviation of infection by improving barrier function	(126)
Parasite infection	Mice	*Trichinella spiralis*	20 mg/kg/day, 2 weeks	Decreased oxidative stress caused by *Trichinella spiralis* infection in small intestine	(134)
Virus infection	Duck	Duck viral enteritis	25 mg/ml	Suppressed the multiplication of duck enteritis virus in host cells	(131)

*NF-κB, nuclear factor kappa B; TNBS, trinitrobenzene sulfonic acid; DSS, dextran sulfate sodium; CACS, chronic-acute combined stress; XO, xanthine oxidase; APEC, avian pathogenic Escherichia coli; IBS, irritable bowel syndrome; DMH, 1,2-dimethylhydrazine, CSC, cancer stem cells*.

### Inflammatory Bowel Disease

Inflammatory bowel diseases (IBD), mainly includes Crohn's disease (CD) and ulcerative colitis (UC), are a group of chronic and relapsing inflammatory diseases in the digestive tract, which could be affected by the genetic, environment, and immune factors. However, it remains to know the precise aetiology of IBD (101). It is worth noting that mucosal injury caused by high level oxidative stress plays a key role in the pathogenesis of IBD, and the activation of nuclear factor κ light-chain-enhancer of activated B cells (NF-κB) also regulates the process of IBD (102). In heat-stressed rats, resveratrol reverses the cortisol level and diamine oxidase (DAO) activity increasing in serum, decreases malondialdehyde (MDA), and increases the mRNA expression of cytokines and antioxidant enzymes (89). Resveratrol (20 mg/kg/day) decreases MDA content, increases glutathione (GSH) level and catalase (CAT) activity in intestine of irradiated rats. Furthermore, resveratrol reduces the contents of inflammatory cytokines, including TNF-α, NF-κB, and IL-1β in intestine (87). In colitis mice, 100 mg/kg resveratrol significantly decreases inflammatory cytokines level of TNF-α, IFN-γ, and IL-17 (90); 2 and 10 mg/kg resveratrol decreases the ulcerative area and colon mass index (88). Likewise, resveratrol suppresses the activation of NF-κB in Caco-2 cells and SW480 human colon cancer cells (103). Sabzevary-Ghahfarokhi et al. (104) used TNF-α-stimulated Caco-2 cells to simulate UC *in vitro*, and it was found that the protein expressions of IL-1β and p-Nrf2 are increased, however, this was reversed by resveratrol treatment. In addition, the destruction of intestinal epithelial cells tight junctions increases the interactions between intestinal microbiota and the host immune system, which also affects the course of IBD (105). In IBD patients, the biodiversity of commensal bacteria is decreased (especially *Bacteroidetes* and *Firmicutes*, including the clinically relevant *Faecalibacterium prausnitzii*); nevertheless, the *Escherichia coli* abundance is increased (106). As mentioned earlier, resveratrol regulates the expression of TJ proteins to improve the physical barrier. In addition, resveratrol regulates the abundance of commensal bacteria and inhibits the growth of *Escherichia coli*. Therefore, it is likely that resveratrol might alleviate IBD.

### Colorectal Cancer

Colorectal cancer (CRC) is one of the three most common cancers in the world, which is a serious threat to human health (107). Chronic inflammation of the intestine is one of the main determinants of CRC. Therefore, anti-inflammatory compounds may play a beneficial role in the treatment of CRC (108). As previously mentioned, resveratrol has anti-inflammation and anti-tumor functions, and it can regulate the proportion of anti-inflammatory and inflammatory immune cells. Moreover, resveratrol decreases pro-inflammatory factors, such as TNF-α and IL-1β, pro-inflammatory enzymes such as iNOS and COX-2 and inflammatory signaling pathways, such as NF-kB. Resveratrol can reduce the number of aberrant crypt foci (ACF) on azoxymethane (AOM)-induced colon carcinogenesis in F344 rats by regulating the expression of Bax and p21 (109). In addition, oxidative stress is also considered to be one of the factors that aggravate cancer, and resveratrol also inhibits lipid peroxidation and scavenge ROS (110, 111). Furthermore, resveratrol has anticancer function mainly through inhibiting proliferation and inducing apoptosis of tumor cell (112, 113). Some studies have found that resveratrol inhibits the tumor cell cycle at the transition S to G2/M which is associated with cyclin and cyclin-dependent kinase activities (114–117). Hence, resveratrol may be a potential compound for the treatment and prevention of CRC. In fact, several studies have shown that resveratrol can ameliorate CRC. Murugan et al. found that resveratrol significantly reduces tumor incidence, histological lesions, and tumor size in 1,2-dimethylhydrazine (DMH)-induced colon cancer of mice, and resveratrol inhibits the proliferation of tumor cells. Resveratrol regulates the oxidative imbalance caused by DMH treatment, which has been shown that the activities of some antioxidant enzymes are increased (92). Yuan et al. (94) shown that resveratrol may suppress the invasion and metastasis of colon cancer through reversal of epithelial mesenchymal transition (EMT) markers *via* the AKT/GSK3β/Snail signaling pathway. Constanze et al. simulated of tumor microenvironment (TME) by multicellular culture *in vitro*, they found that multicellular-TME, similar to TNF-β-TME, promotes proliferation, colony formation, invasion of CRC cells and enables activation of cancer stem cells (CSC). However, resveratrol reduces the secretion of T-lymphocyte/fibroblast (TNF-β, TGF-β3) proteins, antagonizes the T-lymphocyte/fibroblast-promoting NF-κB activation, and NF-κB nuclear translocation. Thus, fibroblasts and T-lymphocytes are promising targets for resveratrol in the prevention of CRC metastasis (93). In addition, in colorectal cancer patients, resveratrol reduces tumor cell proliferation by 5% (91), low dosages of resveratrol combination with other bioactive compounds in freeze-dried grape powder (GP) inhibit Wnt signaling pathway in normal colonic mucosa, that indicated a reduction in the expression of a panel of Wnt target genes, suggesting that resveratrol or GP may play a beneficial role in colon cancer prevention (118).

Altogether, resveratrol can be used as a compound for the treatment or prevention of CRC, perhaps better in combination with chemotherapy. Moreover, resveratrol improves CRC by antagonizing chronic inflammation, oxidative stress, and inhibiting the growth of tumor cells.

### Irritable Bowel Syndrome

Irritable bowel syndrome (IBS) is a functional bowel disorder that causes chronic and recurrent pain (119), and the pathogenesis is still unclear. In recent year, altered gut immune activation, intestinal permeability, and gut microbiome have been identified in some IBS patients (120). Moreover, most IBS patients are accompanied by depression (121), and the Gut-Brain Axis plays an important role in IBS (122). As mentioned earlier, resveratrol could affect microbial and immune barriers. Therefore, we speculate that resveratrol plays an important role in the treatment of IBS. In fact, several studies have shown that resveratrol could ameliorate IBS. Xu et al. utilized chronic-acute combined stress (CACS)-induced IBS-like symptoms (depression, anxiety, and intestinal dysfunction) in ICR male mice, *trans*-resveratrol treatment significantly reverses CACS-induced depression- and anxiety-like behaviors and intestinal dysfunction in mice, including improves hippocampal neuronal remodeling, protects ileal and colonic epithelial barrier structure against CACS insults. The underlying mechanism may be related to normalization of PDE4A expression and CREB-BDNF signaling both in the central nervous and peripheral systems (95). Yu et al. utilized the CACS-induced IBS-like symptoms in male Sprague-Dawley (SD) rats, resveratrol treatment improves anti-IBS-like effects on depression, anxiety, visceral hypersensitivity, and intestinal motility abnormality through regulating 5-HT1A-dependent PKA-CREB-BDNF signaling in the gut-brain axis (96). Thus, resveratrol has an alleviating effect on IBS, and gut-brain axis plays a vital role. In addition, microbiota is the key factor affecting IBS, resveratrol has been shown to affect the intestinal microbial barrier. Thus, resveratrol may also alleviate IBS by affecting the composition of microbiota, and the specific mechanism needs to be further studied.

### Intestinal Infectious Diseases

Some pathogenic microorganisms could cause intestinal infectious diseases and seriously affect intestinal health. As mentioned before, resveratrol inhibits the growth of many pathogens, we speculate that resveratrol can resist intestinal infectious diseases caused by some pathogens. *Escherichia coli* is Gram-negative bacterium as a family member of *Enterobacteriaceae*, which predominantly colonize in intestine of warm-blooded animals such as humans (123). *Escherichia coli* could be divided into commensal and pathogenic strains. The latter [e.g., *enterotoxigenic E. coli* (ETEC), *enterohemorrhagic E. coli* (EHEC), and *enteropathogenic E. coli* (EPEC)] would induce a wide variety of intestinal infections (124). For example, ETEC, the most common cause of bacterial diarrhea, enters the gut and adheres to the small intestinal epithelium through the colonization factors to cause diseases (125). It has been reviewed that the adhesion of pathogenic bacteria on intestinal epithelium is related to the formation of bacteria biofilm (126). Interestingly, resveratrol restrains the growth of avian pathogenic *E. coli* through inhibiting the formation of bacterial biofilm, and the MIC is 128 μg/ml (97). It has been found that the adhesion inhibition of *E. coli O157:H7* to HT-29 colonic cells is more than 60% with resveratrol and its derivatives treatments (127). Overall, the inhibiting adhesion and biofilm formation of *E. coli* on intestinal epithelium might be a target of resveratrol to offer treatment of *E. coli* infection. However, the function of resveratrol on various *E. coli* is still unclear.

*Campylobacter jejuni* (*C. jujuni*) is a pathogen of human, which could cause bacterial diarrheal disease by destroying epithelial barrier, for example, tight-junction disruption and epithelial apoptosis. In addition, occludin and claudin-5 in colonic epithelial cells were redistributed after infection of *C. jujuni* (128). As previous studies shown, resveratrol can attenuate intestinal epithelial damage by *C. jujuni* infection (98) and also alleviate inflammation via decreasing inflammatory factors such as TNF-α or C-reactive protein (CRP) levels (129). However, Lobo et al. shown that the anti-*C. jujuni* effect of resveratrol might base on improving barrier function at the epithelial level instead of decreasing cytokine release. Thus, resveratrol might be a promising compound for the treatment and prevention of *C. jujuni* infection. They also demonstrated that resveratrol can rescues colonic epithelial barrier function through evaluating the intestinal epithelial leakiness in the *C. jujuni*-infected mice (130, 131).

Many studies have stressed the antiviral functions of resveratrol both *in vivo* and *in vitro* (132). Xu et al. (100) has revealed that resveratrol alleviates duck viral enteritis (an acute, contagious and herpesvirus infection of poultry intestine) through suppressing the multiplication of duck enteritis virus in host cells. Meantime, some studies have revealed that resveratrol could inhibit the replication of rotavirus (the main cause of acute severe viral diarrhea of infant animals) in Caco-2 cell lines and ameliorate the severity of diarrhea (133, 134). In addition, resveratrol also plays an important role in the treatment of parasitic infections. Resveratrol could alleviate the oxidative stress caused by *Trichinella spiralis* infection in small intestine (99). Altogether, resveratrol plays an important role in resisting intestinal infection, and the realization of this function depends on the strong antibacterial activity of resveratrol.

## Prospects for Future

In this review, we pay more attention to the therapeutic and preventive effects of resveratrol on intestinal diseases, and we find that this can be achieved by regulating the intestinal barrier integrality. As mentioned earlier, resveratrol does affect the composition of intestinal microorganisms, and resveratrol is considered as a potential prebiotic candidate to promote changes in bacterial composition associated with a healthy phenotype (135). Song et al. (68) found that resveratrol regulates the function of gut microbiota to resist immunosuppression. However, few mechanistic clues as to how the resveratrol-gut microbiota-metabolism axis could be functioning, including resveratrol-gut microbiota-brain axis, resveratrol-gut microbiota-liver axis, and resveratrol-gut microbiota-kidney axis. Thus, resveratrol plays a health-promoting role through the connection between intestinal microorganisms and extraintestinal target organs, which has become a focus of future research. In addition, resveratrol may have another potential mechanism for intestinal barrier modulation. It has been proposed that long non-coding RNAs (lncRNAs) might be a potential regulatory factor for intestinal barrier. Although the influences of lncRNAs are still require a broaden experimental confirmation in intestinal barrier, resveratrol has been reported that it can modulate lncRNAs to combat different diseases, such as insulin resistance (136), lung cancer (137), and prostate cancer (138). Thus, it would be very interesting to explore whether and how resveratrol shapes intestinal barrier via lncRNAs. In addition, a novel experiment has been well demonstrated that the ovarian tumor deubiquitinase 1 (OTUD1) alleviates IBD through inhibiting receptor-interacting serine/threonine-protein kinase 1 (RIPK1)-mediated NF-κB activation (139). Therefore, whether and how resveratrol interacts with OTUD1 to mediate intestinal barrier and ultimately regulate IBD should also be taken into consideration. However, there are many difficulties to overcome with lncRNAs. Firstly, it is still lack studies on the effects of lncRNAs in intestinal barrier. Secondly, it is difficult to select sensitive and specific lncRNAs as clinical biomarkers. Finally, it is still unclear which targeting methods and drug vectors are suitable for lncRNA-targeted therapy (140). Generally, there is still a long way to find more underlying relationships between resveratrol and intestinal barrier.

## Conclusions

**Figure 3:**
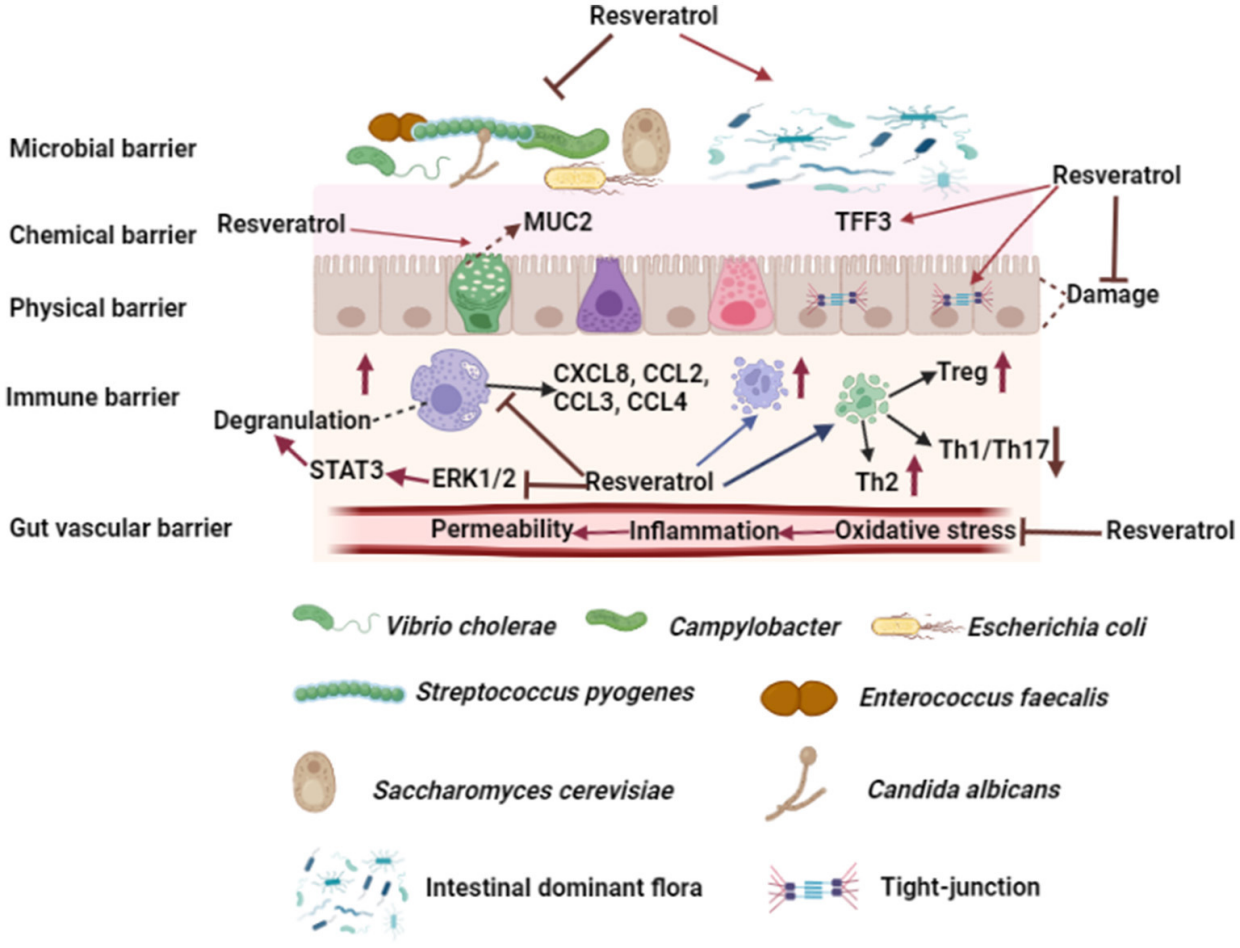
Effects of resveratrol on intestinal barrier. Resveratrol could improve intestinal microbial barrier by inhibiting the growth of pathogens and modulating the composition of intestinal dominant flora. Resveratrol promotes goblet cells to secrete MUC2 and increases TFF3 in mucous layer. Moreover, resveratrol increases the expression of tight junction protein between enterocytes and protects enterocytes from injury. Resveratrol affects T cell differentiation and increases the number of Treg and Th2 cells in intestinal lymph nodes and lamina propria, while decreases the number of Th1 and Th17 cells. Furthermore, resveratrol increases the number of mast cells and macrophages in intestinal lymph nodes and lamina propria, and inhibits mast cell degranulation by inhibiting the phosphorylation of ERK1/2 and STAT3 and affecting mast cell chemokine secretion. Resveratrol improves gut vascular barrier by reducing vascular permeability. The figure was created with Biorender.com.

The intestinal barrier is a complex network, which is composed of microbial, chemical, physical, immune, and vascular barrier. Intestinal barrier not only affects the absorption of nutrients, but also is closely related to body health. Resveratrol is a natural plant polyphenol, which has many biological functions, such as antioxidant, anti-inflammatory, anti-cancer, and cardiovascular protection. In this review, we have introduced the absorption and metabolism of resveratrol by gastrointestinal tract, we also summarize the effects of resveratrol on five intestinal barriers (Figure 3), and it can resist intestinal barrier damage and maintain intestinal barrier function. In addition, resveratrol could treat and/or prevent a variety of intestinal-related diseases, such as IBD, CRC, IBS, and intestinal infectious diseases. In general, our review illustrates that resveratrol can affect intestinal health and disease by regulating the intestinal barrier.

## Author Contributions

YW, CH, ZW, SL, YX, YL, and XH: writing-review and editing. WT: supervision. All authors have read and agreed to the published version of the manuscript.

## Funding

This work was financially supported by the Sichuan Science and Technology Programmes (2021JDYZ0001 and 2021ZDZX0009).

## Conflict of Interest

WT and SL were employed by Sichuan Animtech Feed Co., Ltd. The remaining authors declare that the research was conducted in the absence of any commercial or financial relationships that could be construed as a potential conflict of interest.

## Publisher's Note

All claims expressed in this article are solely those of the authors and do not necessarily represent those of their affiliated organizations, or those of the publisher, the editors and the reviewers. Any product that may be evaluated in this article, or claim that may be made by its manufacturer, is not guaranteed or endorsed by the publisher.
